# Lower Respiratory Tract Infection in Children: When Are Further Investigations Warranted?

**DOI:** 10.3389/fped.2021.708100

**Published:** 2021-07-28

**Authors:** Ziyaad Dangor, Charl Verwey, Sanjay G. Lala, Theodore Mabaso, Keketso Mopeli, Denise Parris, Diane M. Gray, Anne B. Chang, Heather J. Zar

**Affiliations:** ^1^Paediatric Education and Research Ladder, Department of Paediatrics and Child Health, University of the Witwatersrand, Johannesburg, South Africa; ^2^Department of Paediatrics and Child Health, Division of Pulmonology, Chris Hani Baragwanath Academic Hospital, University of the Witwatersrand, Johannesburg, South Africa; ^3^Department of Paeditrics, University of Pretoria, Pretoria, South Africa; ^4^Department of Paediatrics and Child Health, University of Cape Town, Cape Town, South Africa; ^5^South African Medical Research Council Unit on Child and Adolescent Health, University of Cape Town, Cape Town, South Africa; ^6^Department of Respiratory and Sleep Medicine, Queensland Children's Hospital, Queensland University of Technology, Brisbane, QLD, Australia; ^7^Australian Centre for Health Services Innovation, Queensland University of Technology, Brisbane, QLD, Australia; ^8^Child Health Division, Menzies School of Health Research, Charles Darwin University, Darwin, NT, Australia

**Keywords:** LRTI, pneumonia, children, sequelae, lung disease

## Introduction

The substantial decline in the burden of childhood community acquired lower respiratory tract infections (LRTI) over the last decades is associated with improvements in immunization, nutrition, socioeconomic, and control of the HIV epidemic (1). However, LRTI remains the commonest cause of under-5 mortality outside the neonatal period (1). Although most children with LRTI fully recover, a proportion develop chronic respiratory symptoms and/or sequelae; reasons include host factors (immunosuppression, poor secretion clearance, airway abnormalities or genetic factors), infectious causes (TB or adenovirus), and/or adverse environmental factors. Early identification and management of children at-risk of respiratory sequelae may help to preserve long-term lung health. However, knowing who and when to investigate is challenging as there is little high-level evidence to support the timing and extent of investigations required.

In HIV and TB endemic settings, clinicians tend to attribute chronic respiratory symptoms or sequelae to childhood HIV and/or TB; however, improved prevention-of-mother-to-child-transmission (PMTCT) programs and access to antiretroviral therapy (ART) have reduced the burden of infections, including TB (2, 3). Once these infections are excluded, other conditions predisposing to chronic respiratory symptoms and sequelae must be considered (4, 5).

Prolonged, severe or recurrent infection, that may arise from an underlying predisposition, may trigger a vicious cycle of inflammation, impaired mucociliary clearance, and bacterial colonization leading to proteolytic lung damage and bronchiectasis (6, 7). In low- and middle-income countries, and socially disadvantaged communities in high-income countries, post-infectious bronchiectasis is the commonest sequelae of LRTI (8). In sub-Saharan Africa, the causes of childhood bronchiectasis remain poorly defined but the commonest causes are likely to be post-infectious or HIV-associated (9, 10).

This paper aims to provide guidance for healthcare providers to identify, investigate and manage a child with chronic respiratory symptoms (usually cough, wheeze, exercise intolerance) and identify who is at-risk of developing respiratory sequelae (bronchiectasis, post-infectious bronchiolitis obliterans, obstructive and/or restrictive lung function abnormalities, chronic hypoxia, or pulmonary hypertension) after a LRTI episode to minimize the risk of irreversible lung damage (11).

### Diagnosis and Investigation

A single episode of LRTI in a child who fully recovers does not usually warrant further investigation. To decide if investigations are warranted, the SPUR acronym (Box 1) is used to alert the clinician to possible underlying problems (4). Children with “severe” clinical presentations (i.e., prolonged hospitalization, the need for ventilation or ICU admission, and/or multi-system involvement), or the “persistence” of symptoms (slow resolution of LRTI; >2 weeks), or residual symptoms of cough and/or wheeze require further investigation. In these circumstances, first consider: (i) alternate diagnoses, (ii) untreated or resistant pathogens, (iii) host immunosuppression, or (iv) complications such as empyema or abscess (12). In a small proportion of children with LRTI, the presence of “unusual clinical” findings or an “unusual comorbidity” at presentation should prompt further investigation and/or close monitoring. These include: a history of chronic cough or wheeze (generally >4 weeks), chest wall abnormalities, clubbing or pulmonary hypertension; or a comorbidity such as feeding difficulties, HIV, cardiac, or neurological diseases (4, 13, 14). Furthermore, “unusual CXR” or “unusual colony /microbiological findings” may warrant investigation. Lastly, “recurrent” LRTI (generally ≥2 episodes per year) requires further evaluation—an important consideration is whether the LRTI recurs in the same lobe, diffusely or in different areas.

Box 1 The SPUR acronym [adapted from (4)]. ^*^Chronic cough is defined as a cough that is present for at least 4 weeks.

1. Severe
  - Prolonged hospitalization
  - Need for ventilation
  - Multi-system involvement
2. Persistent
  - Clinical – persistence of respiratory symptoms (cough* or wheeze)
  - Radiological – failure of resolution
3. Unusual
  ■ Clinical
    - Chronic upper (otitis-media, rhino-sinusitis) respiratory symptoms
    - History of chronic respiratory symptoms (cough* or wheeze)
    - Poor growth or failure to thrive
    - Feeding difficulties
    - Chest wall abnormalities
      1. Asymmetry
      2. Harrisons sulcus
      3. Pectus excavatum
      4. Pectus carinatum
      5. Barrel shape
    - Digital clubbing
    - Features of pulmonary hypertension
  ■ Co-morbidities
    - HIV-infection
    - Primary or secondary immunodeficiency
    - Malnutrition
    - Neuromuscular disease
    - Cerebral palsy
    - Abnormal neonatal history (prematurity, broncho-pulmonary dysplasia, prolonged ventilation or oxygen dependence)
    - Congenital heart lesion with increased pulmonary blood flow
  ■ CXR
    - Homogenous air space opacification (white-out)
    - Lobar/focal disease
    - Differential transradiancy
    - Airway compression
    - Cystic lucencies
  ■ Colonies
    - e.g.,: *Pseudomonas aeruginosa*, or other opportunistic infections
4. Recurrent

The presence of SPUR “red flags” should prompt the clinician to undertake a thorough history and examination, and consider relevant investigations (Supplementary Figure 1). The history may reveal patterns of symptoms that provide clues to the underlying diagnosis (Supplementary Figure 2).

Firstly, investigate for TB and HIV in high burden settings. TB is a common but often unrecognized cause of LRTI (15). Repeated lower respiratory samples may be needed for microbiological confirmation. Failure to confirm TB or an inadequate response to TB therapy (poor weight gain, and/or persistence or worsening of radiological features) should alert the clinician to consider drug resistant TB, non-adherence to therapy or an alternate diagnosis.

Next consider asthma (in a child with recurrent wheezing, exercise induced dyspnea or nocturnal cough) or protracted bacterial bronchitis (PBB) in an otherwise well-child with a chronic wet cough. In some settings, up to 40% of children with chronic cough may have PBB; untreated PBB increases bronchiectasis risk (16, 17). The definition of PBB is based on the presence of chronic wet cough (>4 weeks), an identified pathogen, and symptom resolution after administering appropriate oral antibiotics for 2 weeks (17).

Most asthma exacerbations are triggered by a viral respiratory tract infection (18). In young children, spirometry is challenging and diagnosing asthma based on bronchodilator responses is difficult. The following features may assist the clinician in diagnosing asthma in young children: (i) recurrent cough or wheezing with activity, or a nocturnal cough; (ii) a first degree relative with asthma; (iii) co-existing eczema or allergic rhinitis; (iv) a clinical response to a 3-month trial of controller therapy; or (v) elevated fractional exhaled Nitric Oxide (FeNO) for age (18). However, these features have a low probability and limited predictive value for asthma (19).

A follow-up chest x-ray is indicated in the presence of ongoing symptoms, lobar collapse, dense consolidation, or recurrence of radiological changes in the same lobe/segment—this may suggest the possibility of bronchial obstruction e.g., congenital airway abnormality, foreign body, lymph node or other cause of external compression. The availability of specialized imaging, lung function testing, a regional pulmonologist, or referral pathways for specialized testing would determine the next steps (Supplementary Figure 1).

Although the quality of evidence is low, the European Respiratory Society Clinical Practice Guideline for managing pediatric bronchiectasis recommended the following first line tests in children with suspected or confirmed bronchiectasis: full blood count, immunologlobulins, sweat test, CT, lung function tests, sampling the lower airway, and assessment for TB and HIV in endemic areas (20). Further immunodefeciency work-up, bronchoscopy, and identification of primary ciliary dyskinesia, gastro-esophageal reflux disease and airway aspiration may be warranted depending on clinical presentation (20).

We recommend using a checklist with an “outside” vs. “inside” lung approach (Table 1). Outside the lung, cardiac (L-R shunts), neuromuscular (weak cough, in-coordinated swallow, bulbar or pseudobulbar palsy), gastrointestinal (tracheo-esophageal fistula, gastro-esophogeal reflux disease), and primary or secondary immunodeficiency conditions should be considered (Table 1). Infants and young children may have up to ten upper respiratory infections per year, more so if they attend crèche or have older siblings (4, 13, 21, 22). Respiratory symptoms after a viral infection can persist for up to 4 weeks, but should get better over time and not be associated with a wet cough or sputum production (4).

**Table 1:** A checklist approach to further investigate children at risk of respiratory sequelae following lower respiratory tract infection.

	**System/site**	**Mechanism/diagnosis**	**Investigation**	**√**
Extra-pulmonary (Outside the lung)	Cardiovascular	Congenital heart disease with increased pulmonary blood flow	ECG Echocardiography	
	Neuromuscular	Weak cough, bulbar or pseudobulbar palsy	Neuro examination	
			Muscle strength testing	
		Dysphagia or swallowing problems with aspiration	Speech assessment	
			Video-fluoroscopy	
	Gastrointestinal	Tracheoesophageal fistula	Contrast Swallow	
			Bronchoscope	
		Reflux disease with aspiration	Video-fluoroscopy	
			Milk scan	
	Upper airway abnormalities	Laryngeal cleft or vocal cord palsy with aspiration	Direct laryngoscopy	
	Immunodeficiency	Nine categories of immune dysfunction (25)	Rule out secondary causes (HIV)	
			Full blood count with differential	
			IgG, IgM, IgA, IgE	
			B and T cell subsets	
			CH50 or CH100	
			Response to vaccines	
			IgG subclasses	
			Lymphocyte proliferation tests	
			Neutrophil Oxidative burst test	
Intra-pulmonary (Within the lung) Congenital or acquired	Compression or obstruction	Outside lumen (vascular anomalies, mediastinal masses—cystic and solid, lymph nodes)	Contrast Swallow	
			CT Chest	
			CT angiogram or MRI	
			Bronchoscope	
		Within lumen (foreign body, bronchial stenosis, tumors, webs, tracheo/ bronchomalacia)	Bronchoscopy	
	Muco-ciliary clearance impairment	Primary ciliary dyskinesia	Nasal brushings or biopsy	
		Cystic fibrosis	Sweat test	
			Fecal elastase	
			Genetic testing	
	Endobronchial suppuration	Protracted bacterial bronchitis or bronchiectasis	CT Chest	
			Sputum for culture	
			Bronchoscopy and lavage	
	Congenital thoracic malformations	Pulmonary sequestration, congenital pulmonary airway malformation (CPAM), lung-agenesis-hypoplasia complex	CT Chest	
	Diffuse lung disease/Childhood interstitial lung disease masquerading as infections	Diffuse developmental disorders, growth, and surfactant abnormalities. Idiopathic interstitial pneumonia. Hemorrhage, eosinophilic, and aspiration syndromes. Hypersensitivity pneumonitis. Connective tissue diseases	CT Chest Bronchoscope and lavage Bloods (ANA, ANCA, etc.) Plethysmography Lung biopsy Diffusion capacity of carbon monoxide	

Within the lung, airway obstruction (outside the wall or within the lumen) or impaired muco-ciliary clearance mechanisms (primary ciliary dyskinesia or cystic fibrosis) result in stasis, bacterial colonization and recurrent inflammation (Supplementary Figure 3). Diffuse/childhood interstitial lung disease masquerading as infection, right middle lobe syndrome, or cystic parenchymal disease are other considerations (Table 1) (4).

### Management

The aim of management is to prevent adverse pulmonary sequelae by early diagnosis and treatment which may interrupt progression to bronchiectasis and lung function impairment. Key management goals for those with bronchiectasis are: to improve quality of life (reduce cough, prevent exacerbations, improve effort tolerance), optimize lung growth (when possible), prevent further lung injury (treat inflammation, prevent exacerbations, treat underlying cause), and minimize complications (20, 23).

Management of at-risk children or those with bronchiectasis (suspected or confirmed) includes (Supplementary Figure 4):

1. General lung protective strategies
  Reduce exposure to environmental insults (smoking or vaping, biomass and fossil gas combustion) that impair physiological processes such as ciliary function. In addition to ensuring that routine childhood immunizations are complete, consider annual influenza vaccination and booster pneumococcal vaccine in at-risk and immunosuppressed children. HIV-infected pregnant women and their infants should receive PMTCT. HIV-infected infants require early initiation of ART, and cotrimaxazole or isoniazid prophylaxis as indicated.
2. Treat the underlying cause
  If identified, treat the underlying cause: eg, remove foreign body.
3. Interrupt the cycle of bacterial infection and inflammation
  a. Airway clearance techniques:
    Age-appropriate chest physiotherapy techniques are a cornerstone of therapy. Percussion of the chest by the caregiver is a feasible technique in younger children whereas self-initiated airway clearance techniques using assisted devices or maneuvers are more practical in older children (20).
    Mucoactive agents such as recombinant-human DNAse, inhaled mannitol or hypertonic saline are not routinely recommended (20). Whilst the latter may be used selectively, recombinant-human DNAse is contraindicated in children without CF (20). Similarly, inhaled corticosteroids and long or short-acting beta2-agonists are not routinely recommended unless children have an asthma phenotype or eosinophilia (20).
  b. Antibiotic therapy can be used for exacerbations, or for eradication and/or control:
    Exacerbations are deviations from baseline and may present with an increased cough frequency, increased sputum production or change of sputum color, and/or the presence of respiratory distress for more than 3 days (20). Antibiotics should be targeted at the organism identified on sputum culture or the colonizing organism identified prior to the exacerbation. Amoxicillin/clavulanic acid (14 days of therapy) is the preferred choice of treatment in children with chronic wet cough, PBB or bronchiectasis (17, 20, 24, 25).
    Eradication and/or control using intravenous, oral or inhaled therapy is necessary for organisms that colonize the airway and cause long-term deterioration in lung function, e.g., *Pseudomonas aeruginosa* (20).
  c. Anti-inflammatory agents:
    Azithromycin may be considered on alternate days for a minimum of 6 months (20). One RCT has shown that a long-term macrolide, azithromycin (10 mg per kg; max 500 mg), halves exacerbation rates in children with non-CF bronchiectasis (incidence rate ratio 0.50; 95% CI 0.35–0.71 compared to placebo) (26). The data in children is similar to the effect size described in meta-analyses of adults with bronchiectasis (27).
4. *Monitoring for disease progression* is essential. The following parameters should be monitored at least 6-monthly:
  a. Clinical monitoring of cough, exercise intolerance, sputum color, frequency of exacerbations, co-morbidities and complications.
  b. Microbiological monitoring of sputum to identify the colonizing organism is essential to tailor antimicrobial therapy during exacerbations, and eradicate/control unwanted colonizers such as *Pseudomonas aeruginosa* (20).
  c. Spirometry or other lung function is required to document stability (20).
  d. Radiological monitoring is usually done dependent on clinical features, and resource limitations (20). There is limited evidence to support baseline and repeat low radiation dose CT scans in resource limited settings.
5. Other supportive measures
  Adequate nutrition is essential for lung growth in the first few years and important throughout life. There is no evidence to support the use of nutritional supplements except for Vitamin D supplements in deficient children (20). Concurrent gastro-esophageal reflux disease or asthma should be appropriately managed (18). Furthermore, traditional or cultural remedies that cause lipoid pneumonia should be specifically sought in setting where these practices are common (28). In children with severe disease and lung destruction, supplementary domiciliary oxygen and treatment of pulmonary hypertension and right heart failure may be required.

## Conclusion

Clinicians should maintain a high level of suspicion for children at-risk of developing chronic respiratory symptoms or sequelae following LRTI. In endemic settings, TB and/ or HIV infection should be diagnosed or excluded promptly. Thereafter, other conditions that may lead to the development of chronic respiratory symptoms or sequelae should be considered. Early recognition of underlying disease and appropriate management can prevent disease progression, improve quality of life and optimize lung function.

## Author Contributions

ZD prepared the first draft. All authors contributed to the article and approved the submitted version.

## Conflict of Interest

The authors declare that the research was conducted in the absence of any commercial or financial relationships that could be construed as a potential conflict of interest.

## Publisher's Note

All claims expressed in this article are solely those of the authors and do not necessarily represent those of their affiliated organizations, or those of the publisher, the editors and the reviewers. Any product that may be evaluated in this article, or claim that may be made by its manufacturer, is not guaranteed or endorsed by the publisher.

## Supplementary Material

Supplementary Figure 1 Pathway for children that require further investigation.

Supplementary Figure 2 Patterns of lower respiratory tract symptoms over a 12 month period [Adapted Penny ME. Pediatr Infect Dis. (1993) 12:762–3].

Supplementary Figure 3 Infographic demonstrating how airway obstruction by a foreign body **(B)** or impairment of muco-ciliary mechanism **(C)** result in stasis of secretions and inflammation as compared to normal **(A)**.

Supplementary Figure 4 Managment steps of children with chronic respiratory symptoms or at-risk of respiratory sequelae.
