# Supplementary figures and images for: Lower Respiratory Tract Infection in Children: When Are Further Investigations Warranted?
Source: Front Pediatr. 2021 Jul 28;9:708100. doi: 10.3389/fped.2021.708100 (PMC8356913; doi:10.3389/fped.2021.708100)

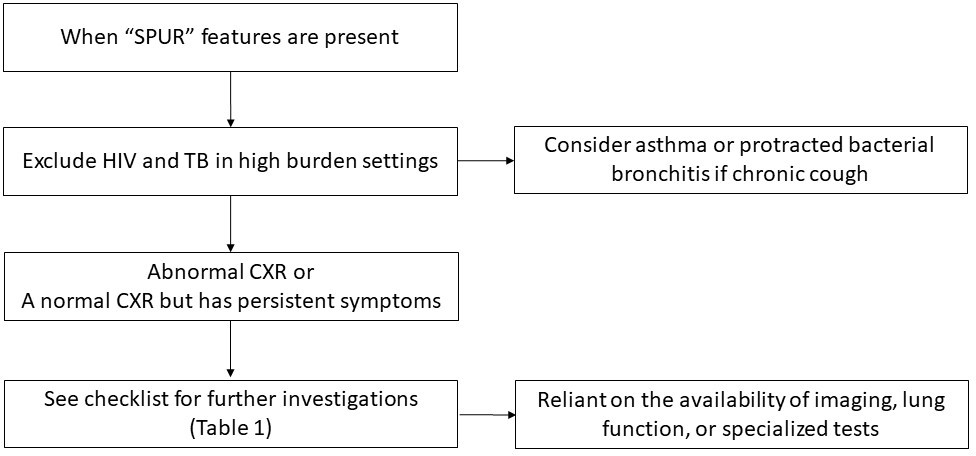

Supplement: Supplementary Figure 1 — Pathway for children that require further investigation. [file Image_1.JPEG]

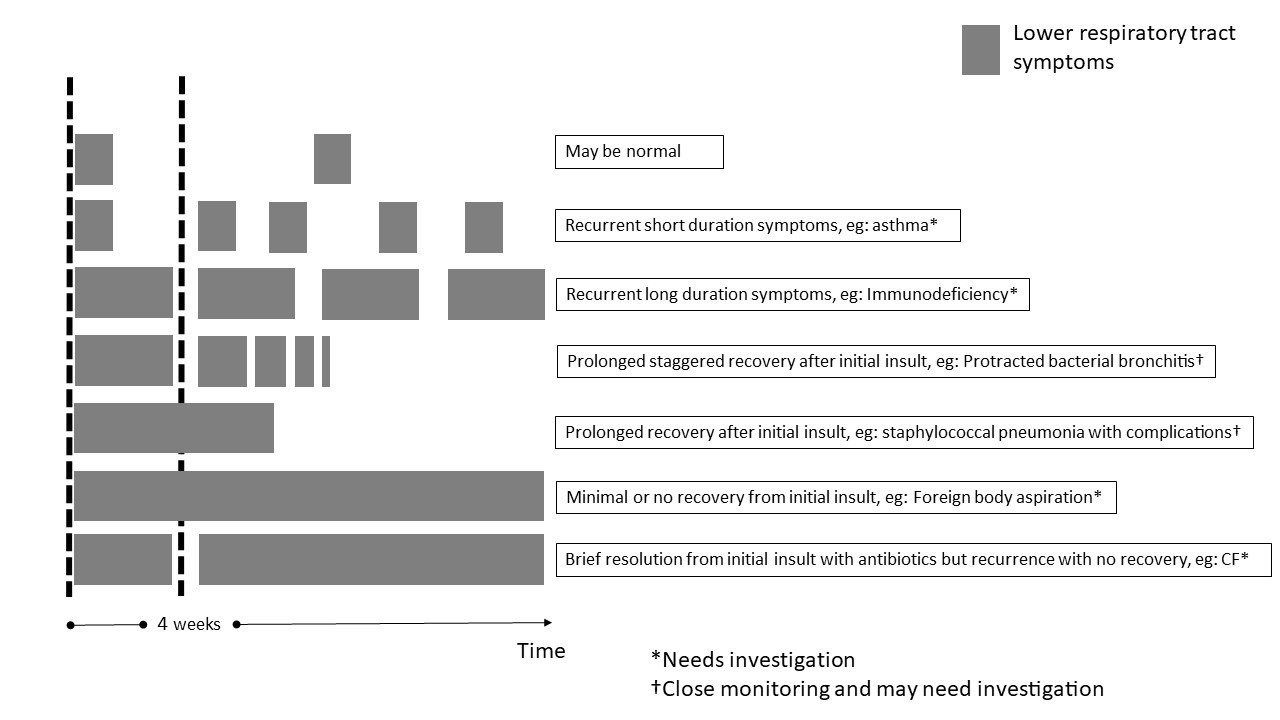

Supplement: Supplementary Figure 2 — Patterns of lower respiratory tract symptoms over a 12 month period [Adapted Penny ME. Pediatr Infect Dis. (1993) 12:762–3]. [file Image_2.JPEG]

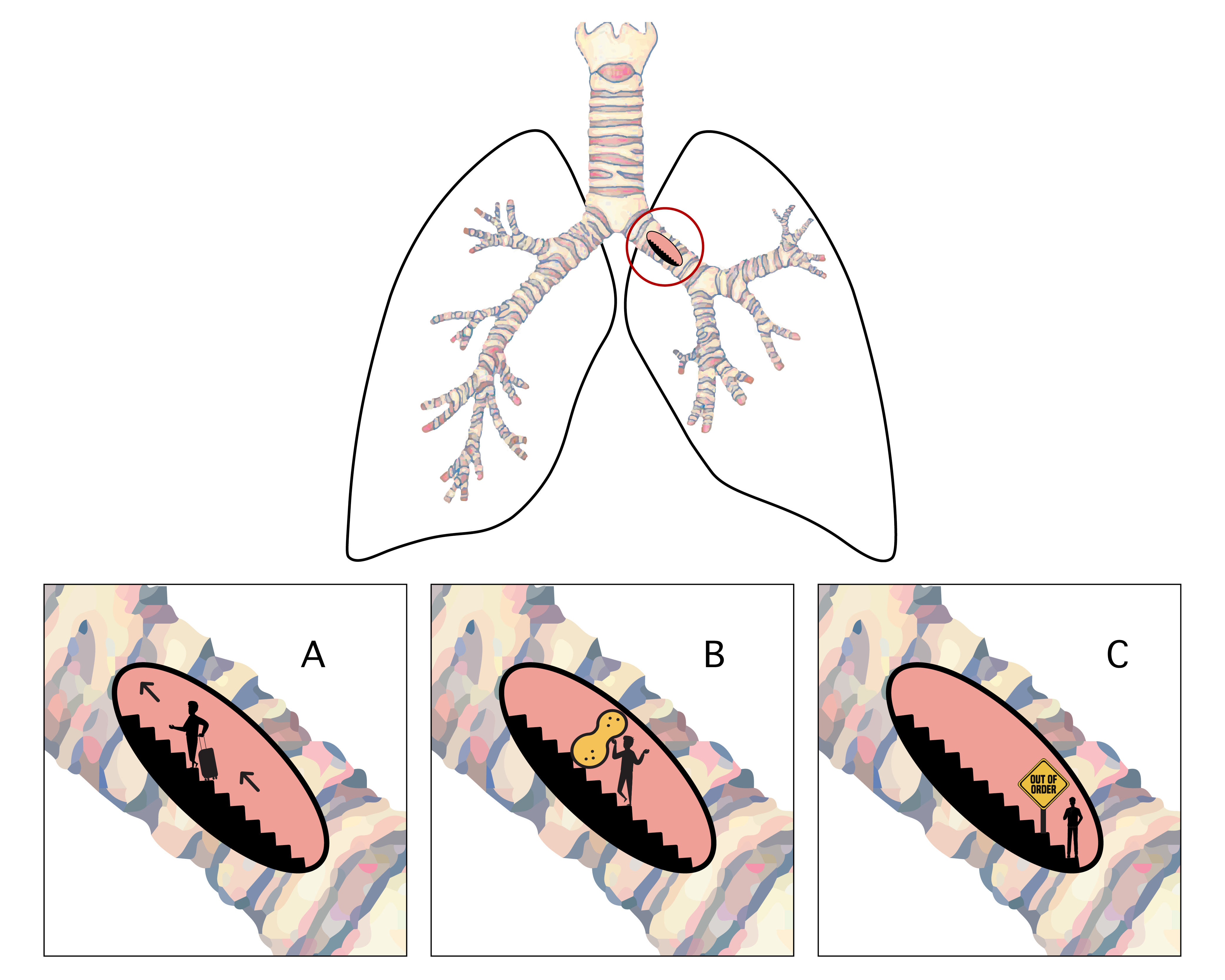

Supplement: Supplementary Figure 3 — Infographic demonstrating how airway obstruction by a foreign body (B) or impairment of muco-ciliary mechanism (C) result in stasis of secretions and inflammation as compared to normal (A). [file Image_3.JPEG]
