# Supplementary material for: Lower Respiratory Tract Infection in Children: When Are Further Investigations Warranted?
Source: Front Pediatr. 2021 Jul 28;9:708100. doi: 10.3389/fped.2021.708100 (PMC8356913; doi:10.3389/fped.2021.708100)
Supplement: Supplementary Figure 4 — Managment steps of children with chronic respiratory symptoms or at-risk of respiratory sequelae. [file Image_4.PDF]

General lung protective strategies

- Immunizations:
  - Routine & catch-up immunizations up to date
  - Booster pneumococcal vaccine
  - Annual influenza vaccine
- SARS-CoV-2: Reduce risk of transmission or infection by social distancing, use of masks, good ventilation and widespread vaccination
- Reduce HIV infection and exposure, and treat appropriately
- Avoid tobacco smoke exposure from antenatal period onwards
- Minimize exposure to indoor and outdoor air pollution

Regular review, monitor & treatment of underlying disease

Interrupt the cycle of bacterial infection and inflammation

- Teach home-based physiotherapy to facilitate airway clearance
- Prescribe antibiotics for acute exacerbations or to eradicate/ control pathogens
- Consider azithromycin for specific cases

Other supportive measures

- Promote adequate nutrition, general hygiene, and physical activity
- Treat associated asthma/ gastro-esophageal reflux disease if present
- Ensure uninterrupted supply of domiciliary oxygen if required
- Treat complications such as pulmonary hypertension or right heart failure

Follow up instructions

- Schedule clinical reviews every 4-6 months in stable children, or more frequently in children currently undergoing investigation, or those with uncontrolled disease
- Every 6-months:
  - Obtain sputum for microscopy and culture
  - Perform spirometry in children who are able to
